# Predicting the Efficacy of Adjuvant Chemotherapy Using Immuno‐Nutritional and Inflammatory Markers in Elderly Patients With Stage III Colorectal Cancer

**DOI:** 10.1002/ags3.70235

**Published:** 2026-06-14

**Authors:** Tomoya Tago, Yasuyuki Takamizawa, Takeharu Kato, Hiroshi Nagata, Konosuke Moritani, Shunsuke Tsukamoto, Yukihide Kanemitsu

**Affiliations:** ^1^ Department of Colorectal Surgery National Cancer Center Hospital Tokyo Japan; ^2^ Department of Gastrointestinal and Pediatric Surgery Tokyo Medical University Tokyo Japan

**Keywords:** adjuvant chemotherapy, colorectal cancer, elderly, marker

## Abstract

**Aim:**

Use of postoperative adjuvant chemotherapy for colorectal cancer (CRC) in elderly patients raises concerns regarding efficacy and toxicity. This study aimed to determine whether immuno‐nutritional and inflammatory markers could serve as predictors of the outcomes of postoperative adjuvant chemotherapy in this population.

**Methods:**

Patients aged ≥ 70 years who were diagnosed with stage III CRC and treated at the National Cancer Center Hospital in Japan between 2000 and 2017 were retrospectively analyzed. Long‐term survival outcomes were compared after propensity score matching (PSM) according to whether adjuvant chemotherapy was administered. Subgroup analyses were performed to determine the interaction of immuno‐nutritional and inflammatory markers with the outcomes of postoperative adjuvant chemotherapy.

**Results:**

A total of 606 patients were included, and PSM resulted in 78 matched pairs. After PSM, there were significant improvements in recurrence‐free survival (RFS) and overall survival (OS) in the group that received adjuvant chemotherapy (RFS: Hazard ratio (HR) 0.537, 95% confidence interval (CI) 0.302–0.955, *p* = 0.034; OS: HR 0.495, 95% CI 0.247–0.990, *p* = 0.047, respectively). The postoperative lymphocyte‐to‐monocyte ratio (LMR) significantly affected RFS and OS (interaction *p* = 0.030 and interaction *p* = 0.023, respectively), and the postoperative C‐reactive protein‐to‐albumin ratio (CAR) significantly affected RFS (interaction *p* = 0.033).

**Conclusion:**

Postoperative LMR and postoperative CAR were associated with the outcome of adjuvant chemotherapy and may help inform its administration in elderly patients with stage III CRC.

## Graphical abstract

This study aimed to determine whether immuno‐nutritional and inflammatory markers could serve as predictors of the outcomes of postoperative adjuvant chemotherapy for stage III colorectal cancer in elderly patients. Postoperative lymphocyte‐to‐monocyte ratio and postoperative C‐reactive protein‐to‐albumin ratio accurately predicted the efficacy of adjuvant chemotherapy and could guide its administration.

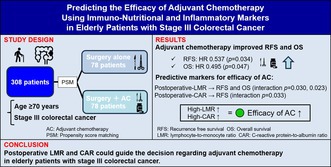

## 1 Introduction

Colorectal cancer (CRC) is one of the most common malignancies in terms of morbidity and mortality worldwide [1]. Surgery is very effective for CRC, particularly in the absence of distant metastasis, and radical resection is the first choice. However, stage III CRC is associated with lymph node metastasis and has a high recurrence rate of 20%–30% [2]. The efficacy of adjuvant chemotherapy has been established by randomized controlled trials, and it plays a crucial therapeutic role in improving survival in colorectal cancer [3, 4].

With advancing age beyond 70 years, the prevalence of comorbidities increases and organ function declines, as previously reported [5]. In general, elderly patients are at a disadvantage with respect to chemotherapy tolerability and the risk of adverse events. In addition, in patients aged ≥ 70 years, there is no consistent evidence supporting an additional benefit of oxaliplatin in adjuvant chemotherapy, suggesting that the therapeutic effect of adjuvant chemotherapy may be limited in this age group [6]. Therefore, identifying subgroups of elderly patients aged ≥ 70 years who are most likely to benefit from adjuvant chemotherapy is of clear clinical importance.

In recent years, immuno‐nutritional and inflammatory markers have been widely reported to be useful for predicting cancer prognosis, postoperative complications, and the efficacy and tolerability of chemotherapy [7, 8, 9, 10]. Several studies have identified poor prognostic factors for adjuvant chemotherapy, including a low preoperative platelet‐to‐lymphocyte ratio (PLR), a low preoperative lymphocyte‐to‐monocyte ratio (LMR), and a low postoperative C‐reactive protein (CRP)‐to‐albumin ratio (CAR) [11, 12, 13]. Although these findings were derived from studies involving patients across all age groups, they may also be extrapolated to elderly patients. Furthermore, cancer progression is influenced not only by tumor‐related factors but also by host‐related responses, and both are known to play critical and interdependent roles. Changes in tumor status and surgical stress can alter the systemic and local immune‐inflammatory responses of both the tumor and the host, which interact with each other; therefore, the clinical significance of immune‐inflammatory markers is considered to differ substantially between the preoperative and postoperative settings.

Accordingly, this study aimed to focus exclusively on elderly patients with stage III CRC who underwent curative resection and to comprehensively analyze preoperative and postoperative immuno‐nutritional and inflammatory markers within the same cohort, in order to clarify their association with the efficacy of postoperative adjuvant chemotherapy.

## 2 Methods

### 2.1 Subjects and Criteria

We retrospectively analyzed consecutive patients aged ≥ 70 years who were diagnosed with CRC without distant metastasis and received surgical treatment in the Department of Colorectal Surgery at the National Cancer Center Hospital in Japan between January 2000 and December 2017. Patients who had undergone radical resection and had pathologically confirmed stage III CRC with adenocarcinoma were included. Patients with appendiceal cancer were excluded, as were those with other synchronous or metachronous primary cancers, those with metachronous CRC excluding intraepithelial cancers within 5 years before surgery, those who received neoadjuvant chemotherapy or adjuvant radiotherapy, and those without complete pre/postoperative data except for missing values related to the controlling nutritional status (CONUT) score.

### 2.2 Treatment and Follow‐Up

All patients were treated and followed up after surgery in accordance with the 2019 Japanese Society for Cancer of the Colon and Rectum guidelines [14]. Treatment for resectable CRC without distant metastases is radical resection based on the concept of complete or total mesorectal excision. In patients with rectal cancer, neoadjuvant chemotherapy, radiotherapy, or chemoradiotherapy is not routinely provided, and lateral lymph node dissection is indicated when the lower border of the tumor is located distal to the peritoneal reflection and if the tumor has invaded beyond the muscularis propria [14, 15]. Adjuvant chemotherapy is administered within 4–8 weeks after surgery in patients with positive lymph node metastases [14].

All the patients in this study were indicated for postoperative adjuvant chemotherapy, but in practice each physician decided whether to provide it based on the patient's age, comorbidities, Eastern Cooperative Oncology Group performance status, and the patient's wishes. The treatment era also determined the aggressiveness of adjuvant chemotherapy in elderly patients. The 2000–2008 period was a time of transition regarding the concept of adjuvant chemotherapy for the elderly, with no guidance from the Japanese Society for Cancer of the Colon and Rectum available, and the indication was determined on an individual basis rather than using age as a limiting factor for treatment. Since 2009, the policy has been to consider adjuvant chemotherapy for elderly patients, and this has been clearly stated in the Japanese Society for Cancer of the Colon and Rectum guideline [16]. Recently, it has been strongly recommended that patients aged > 70 years receive adjuvant chemotherapy provided that they have good performance status, major organ function is preserved, and there are no underlying diseases or comorbidities that would pose a risk if chemotherapy is administered [14]. Furthermore, oxaliplatin was not covered by insurance for adjuvant chemotherapy in CRC until August 2009. Since then, the Japanese guidelines have required that the decision to use oxaliplatin as adjuvant chemotherapy in elderly patients should be made carefully based on age, comorbidities, and the smaller expected benefits [14].

The regimen was either single‐agent therapy with a fluoropyrimidine or combination therapy with a fluoropyrimidine and oxaliplatin for 6 months. Physicians could select from 5‐fluorouracil (5‐FU), 5‐FU + levo‐leucovorin, tegafur/uracil, tegafur/uracil + leucovorin, S‐1, capecitabine, CAPOX (capecitabine and oxaliplatin), and FOLFOX (5‐FU, leucovorin, and oxaliplatin). Regular follow‐up was provided for up to 5 years after radical resection (every 3 months in years 1–3, every 6 months in years 4–5) with clinical examination, laboratory investigations, including carcinoembryonic antigen (CEA) and carbohydrate antigen 19–9 (CA19‐9) every 3 or 6 months, computed tomography of the chest, abdomen, and pelvis every 6 months and colonoscopy every 2 years.

### 2.3 Data Collection

The following demographic, clinical, and pathological data were obtained from the medical records in our departmental database: sex, age, body mass index (BMI), location of the primary tumor (colon or rectum), comorbidities, T and N categories according to the Union for International Cancer Control TNM classification (eighth edition) based on the pathological diagnosis and histology (“differentiated” was defined as tubular adenocarcinoma and papillary adenocarcinoma and “other” as poorly differentiated adenocarcinoma, mucinous adenocarcinoma, or signet‐ring cell carcinoma) [17]. Comorbidity was defined as present if there were any one of the following: hypertension, cardiac disease, cerebrovascular disease, respiratory disease, liver dysfunction, renal dysfunction, and diabetes mellitus. The preoperative laboratory data, including CEA and CA19‐9 levels, were obtained at the first visit to our hospital or immediately before surgery and the postoperative data were obtained at the first visit after surgery between 4 and 8 weeks. The following immuno‐nutritional and inflammatory markers were calculated from the pre‐ and postoperative laboratory data; neutrophil‐to‐lymphocyte ratio (NLR), LMR, PLR, CAR, prognostic nutritional index (PNI), geriatric nutritional risk index (GNRI), CONUT score, Glasgow Prognostic Score (GPS), and modified GPS. NLR was defined as the absolute neutrophil count divided by the absolute lymphocyte count, LMR as the absolute lymphocyte count divided by the absolute monocyte count, and PLR as the absolute platelet count divided by the absolute lymphocyte count. CAR was defined as the CRP concentration (mg/dL)/albumin concentration (g/dL) ratio [13]. PNI was defined as 10× the albumin concentration (g/dL) + 0.005 × the total lymphocyte count (per mm^3^) [18]. GNRI was defined as 1.489 × albumin (g/L) + 41.7 × (weight/WLo), and (weight/WLo) was substituted by the BMI/22 ratio [19]. CONUT scores were calculated summing scores for serum albumin, total peripheral lymphocyte count, and total cholesterol [20]. Albumin concentrations ≥ 3.5, 3.0–3.49, 2.5–2.99, and < 2.5 g/dL were scored as 0, 2, 4, and 6 points, respectively, total lymphocyte counts ≥ 1600, 1200–1599, 800–1199, and < 800/mm^3^ as 0, 1, 2, and 3 points, and total cholesterol concentrations ≥ 180, 140–179, 100–139, and < 100 mg/dL as 0, 1, 2, and 3 points. The GPS was scored as follows: 0, CRP ≤ 1.0 mg/dL and albumin ≥ 3.5 g/dL; 1, CRP > 1.0 mg/dL or albumin < 3.5 g/dL; and 2, CRP > 1.0 mg/dL and albumin < 3.5 g/dL [21]. The modified GPS was scored as follows: score 0, CRP ≤ 1.0 mg/dL; score 1, CRP > 1.0 mg/dL and albumin ≥ 3.5 g/dL; and score 2, CRP > 1.0 mg/dL and albumin < 3.5 g/dL [22]. Each calculation method was based on a previous report.

### 2.4 Statistical Analysis

Fisher's exact test was used for categorical variables and the Mann–Whitney *U* test for continuous variables when comparing differences in patient characteristics and pre/postoperative immuno‐nutritional and inflammatory markers. Analyses were performed for recurrence‐free survival (RFS) and overall survival (OS). RFS was calculated as the interval between the date of surgery and the date of detection of recurrence or death, and OS as the interval between the date of radical resection and the date of death from any cause. Appropriate cut‐off values for the markers represented by continuous variables were calculated by receiver‐operating characteristic curve analysis with 5‐year postoperative RFS and OS as outcomes. The cumulative survival rate was calculated by Kaplan–Meier survival curve analysis, and differences between groups were examined using the log‐rank test. Survivors were censored at the data cut‐off date (April 1, 2023). Univariable Cox proportional hazard regression analysis was used to calculate hazard ratio (HR) as relative risks with corresponding 95% confidence intervals (CI). The interaction between the markers and the effect of postoperative adjuvant chemotherapy on survival was evaluated by multivariable Cox proportional hazard regression analysis. We considered the possibility of bias regarding selection of older patients for adjuvant chemotherapy in view of the greater likelihood of individual differences in background characteristics in this age group. In order to reduce this source of bias, propensity score matching (PSM) was performed to adjust the covariates between patients who received adjuvant chemotherapy and those who did not. PSM included the following variables: sex (male/female), age, BMI, comorbidities (yes/no), location of the primary tumor (colon or rectum), T category (T1–2/T3–4), N category (N1/N2), histology (well differentiated, moderately differentiated, papillary/poorly differentiated, mucinous, or signet‐ring cell carcinoma), preoperative CEA and CA19‐9 levels. PSM was performed using a one‐to‐one nearest neighbor matching algorithm without replacement and a caliper value equal to 0.2 of the standard deviation of the propensity scores. Cut‐off values for each marker were determined using the entire cohort before PSM to avoid bias related to post‐matching data dependency.

All statistical analyses were performed using Statistical Package for Social Sciences software version 27.0 (IBM Corp, Statistics for Windows, Armonk, NY, USA) and EZR software version 1.65 (Saitama Medical Center, Jichi Medical University, Saitama, Japan). All statistical tests were two‐sided, and a *p* < 0.05 was considered statistically significant.

## 3 Results

### 3.1 Patient Selection and Characteristics

**FIGURE 1:**
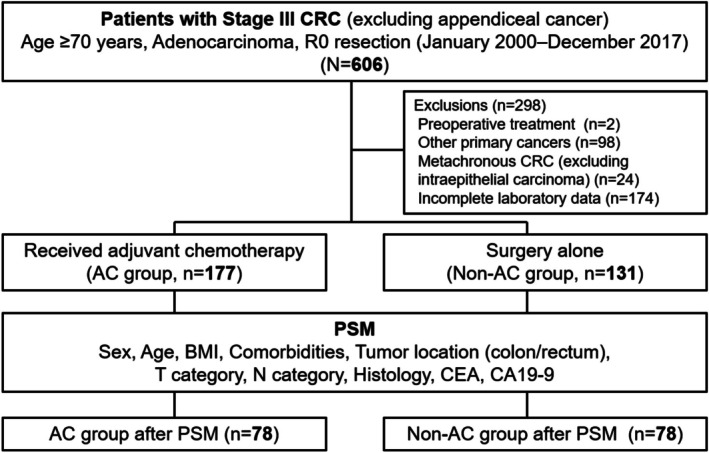
Patient flow diagram. After propensity score matching, data for 156 patients were available for analysis. *AC* adjuvant chemotherapy, *BMI* body mass index, *CA19‐9* carbohydrate antigen 19–9, *CEA* carcinoembryonic antigen, *CRC* colorectal cancer, *PSM* propensity score matching.

Initially, 606 patients aged ≥ 70 years who had undergone radical resection and were diagnosed pathologically to have stage III CRC with adenocarcinoma were identified. The median follow‐up period was 63.0 months (range, 1.5–157.6). We excluded patients who had received preoperative treatment (*n* = 2), those with synchronous or metachronous primary cancers (*n* = 98), those who had developed metachronous CRC within 5 years before surgery excluding intraepithelial cancers (*n* = 24), and those with incomplete pre/postoperative data (*n* = 174). Of the 308 patients who remained, 177 received postoperative adjuvant chemotherapy (the AC group) and 131 did not (the non‐AC group). Patients in the AC group were significantly younger (*p* < 0.001). After PSM, there were 78 pairs of patients in each group, with acceptable differences and balance in background characteristics between them. A patient flow diagram is shown in Figure 1 and a summary of the patient characteristics before and after PSM is shown in Table 1.

**TABLE 1:** Patient characteristics.

Before PSM						
Variable	Category	Total (*N* = 308)	AC group (*n* = 177)	Non‐AC group (*n* = 131)	*P*	SMD
Sex	Male	148 (48.1%)	83 (46.9%)	65 (49.6%)	0.646	0.055
	Female	160 (51.9%)	94 (53.1%)	66 (50.4%)		
Age, years	Median	74 (70–92)	73 (70–86)	78 (70–92)	< 0.001	0.897
	70–74	159 (51.6%)	117 (66.1%)	42 (32.1%)	< 0.001	
	75–79	86 (27.9%)	50 (28.2%)	36 (27.5%)		
	≥ 80	63 (20.5%)	10 (5.7%)	53 (40.4%)		
BMI	Median	22.24 (14.64–36.39)	22.49 (15.25–32.8)	21.82 (14.64–36.39)	0.053	0.224
Comorbidities	Yes	113 (36.7%)	61 (34.5%)	52 (39.7%)	0.403	0.108
	No	195 (63.3%)	116 (65.5%)	79 (60.3%)		
Location	Colon	203 (65.9%)	113 (63.8%)	90 (68.7%)	0.397	0.103
	Rectum	105 (34.1%)	64 (36.2%)	41 (31.3%)		
pT	T1–2	51 (16.6%)	32 (18.1%)	19 (14.5%)	0.441	0.097
	T3–4	257 (83.4%)	145 (81.9%)	112 (85.5%)		
pN	N1	235 (76.3%)	129 (72.9%)	106 (80.9%)	0.106	0.192
	N2	73 (23.7%)	48 (27.1%)	25 (19.1%)		
Histology	well‐mod, pap	286 (92.9%)	168 (94.9%)	118 (90.1%)	0.120	0.184
	por, muc, sig	22 (7.1%)	9 (5.1%)	13 (9.9%)		
CEA	Normal	248 (80.5%)	144 (81.4%)	104 (79.4%)	0.666	0.050
	Elevated (> 5 U/mL)	60 (19.5%)	33 (18.6%)	27 (20.6%)		
CA19‐9	Normal	236 (76.6%)	143 (80.8%)	93 (71.0%)	0.056	0.231
	Elevated (> 37 U/mL)	72 (23.4%)	34 (19.2%)	38 (29.0%)		

After PSM						
Variable	Category	Total (*N* = 156)	AC group (*n* = 78)	Non‐AC group (*n* = 78)	*P*	SMD
Sex	Male	79 (50.6%)	39 (50.0%)	40 (51.3%)	1.000	0.026
	Female	77 (49.4%)	39 (50.0%)	38 (48.7%)		
Age, years	Median	74 (70–89)	74 (70–86)	74 (70–89)	0.581	0.021
	70–74	86 (55.1%)	44 (56.4%)	42 (53.8%)	0.705	
	75–79	50 (32.1%)	26 (33.3%)	24 (30.8%)		
	≥ 80	20 (12.8%)	8 (10.3%)	12 (15.4%)		
BMI	Median	22.49 (14.64–36.39)	22.46 (15.33–32.28)	22.52 (14.64–36.39)	0.877	0.011
Comorbidities	Yes	43 (27.6%)	17 (21.8%)	26 (33.3%)	0.151	0.260
	No	113 (72.4%)	61 (78.2%)	52 (66.7%)		
Location	Colon	95 (60.9%)	45 (57.7%)	50 (64.1%)	0.512	0.132
	Rectum	61 (39.1%)	33 (42.3%)	28 (35.9%)		
pT	T1–2	25 (16.0%)	12 (15.4%)	13 (16.7%)	1.000	0.035
	T3–4	131 (84.0%)	66 (84.6%)	65 (83.3%)		
pN	N1	119 (76.3%)	58 (74.4%)	61 (78.2%)	0.707	0.091
	N2	37 (23.7%)	20 (25.6%)	17 (21.8%)		
Histology	well‐mod, pap	148 (94.9%)	72 (92.3%)	76 (97.4%)	0.276	0.234
	por, muc, sig	8 (5.1%)	6 (7.7%)	2 (2.6%)		
CEA	Normal	127 (81.4%)	63 (80.8%)	64 (82.1%)	1.000	0.033
	Elevated (> 5 U/ml)	29 (18.6%)	15 (19.2%)	14 (17.9%)		
CA19‐9	Normal	122 (78.2%)	63 (80.8%)	59 (75.6%)	0.561	0.124
	Elevated (> 37 U/ml)	34 (21.8%)	15 (19.2%)	19 (24.4%)		

*Note:* Data are presented as the number (percentage) or median (range) as appropriate.

Abbreviations: AC, adjuvant chemotherapy; BMI, body mass index; CA19‐9, carbohydrate antigen 19–9; CEA, carcinoembryonic antigen; PSM, propensity score matching; SMD, standardized mean differences.

### 3.2 Adjuvant Chemotherapy

The 177 patients in the AC group received the following adjuvant chemotherapy regimens: 5‐FU (*n* = 2, 1.1%), 5‐FU + levo‐leucovorin (*n* = 26, 14.6%), tegafur/uracil (*n* = 7, 4.0%), tegafur/uracil + leucovorin (*n* = 21, 11.9%), S‐1 (*n* = 15, 8.4%), capecitabine (*n* = 92, 52.0%), CAPOX (*n* = 7, 4.0%), and FOLFOX (*n* = 7, 4.0%). Before PSM, 163 patients (92.1%) received a single 5‐FU‐based regimen and 14 (7.9%) received an oxaliplatin‐containing double regimen as adjuvant chemotherapy; after PSM, 76 (97.4%) received a single regimen and 2 (2.6%) received a double regimen. When stratified by treatment era, adjuvant chemotherapy was administered to 51 of 102 patients (50.0%) treated up to the end of 2008 and to 126 of 206 patients (61.2%) treated from 2009 onward (*p* = 0.067).

### 3.3 Survival Analysis

**FIGURE 2:**
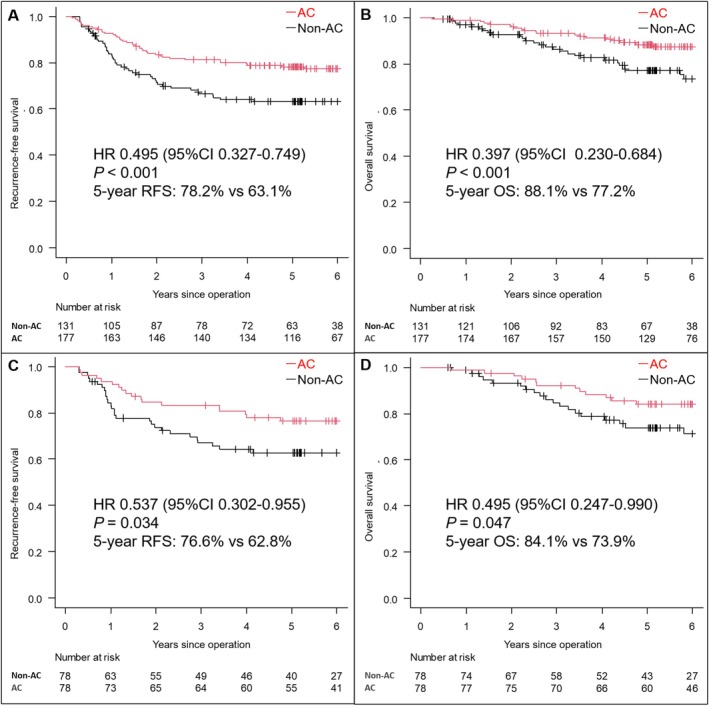
Kaplan–Meier survival curves after propensity score matching according to whether or not adjuvant postoperative chemotherapy was administered. (A) RFS before PSM. (B) OS before PSM. (C) RFS after PSM. (D) OS after PSM. *AC* adjuvant chemotherapy, CI confidence interval, HR hazard ratio, *OS* overall survival, *PSM* propensity score matching, *RFS* recurrence‐free survival.

Before PSM, there were significant differences in RFS and OS between the AC group and the non‐AC group (RFS: HR 0.495, 95% CI 0.327–0.749, *p* < 0.001; OS: HR 0.397, 95% CI 0.230–0.684, *p* < 0.001). The 5‐year RFS rates for the AC and non‐AC groups were 78.2% and 63.1%, respectively, and the 5‐year OS rates were 88.1% and 77.2% (Figure 2A,B). After PSM, significant differences in RFS and OS remained between the AC and non‐AC groups (RFS: HR 0.537, 95% CI 0.302–0.955, *p* = 0.034; OS: HR 0.495, 95% CI 0.247–0.990, *p* = 0.047). The 5‐year RFS rates for the AC and non‐AC groups were 76.6% and 62.8%, respectively, and the 5‐year OS rates were 84.1% and 73.9% (Figure 2C,D).

### 3.4 Characteristics and Cut‐Off Values for Immuno‐Nutritional and Inflammatory Markers

The NLR, LMR, PLR, CAR, PNI, GNRI, CONUT, GPS, and modified GPS values were compared between the AC group and the non‐AC group at both the preoperative and postoperative time points (Table 2).

**TABLE 2:** Nutritional and inflammatory markers.

Before surgery					
Marker	Category	Total (*N* = 308)	AC group (*n* = 177)	Non‐AC group (*n* = 131)	*p*
NLR		2.40 (0.95–11.76)	2.29 (1.00–11.76)	2.53 (0.95–8.67)	0.070
LMR		5.18 (1.08–14.38)	5.49 (1.56–14.39)	4.67 (1.08–14.29)	< 0.001
PLR		151.9 (29.0–575.5)	141.4 (67.1–419.1)	159.3 (29.0–575.5)	0.072
CAR		0.026 (0.002–4.677)	0.025 (0.002–1.608)	0.041 (0.004–4.677)	< 0.001
PNI		49.4 (31.8–64.5)	50.3 (31.8–63.1)	47.9 (32.5–64.5)	< 0.001
GNRI		103.9 (77.1–126.6)	105.1 (79.2–126.6)	101.0 (77.1–121.1)	< 0.001
CONUT score	0–1	192 (63.1%)	122 (69.7%)	70 (54.3%)	0.004
	2–4	102 (33.6%)	51 (29.1%)	51 (39.5%)	
	≥ 5	10 (3.3%)	2 (1.2%)	8 (6.2%)	
	Unknown	4	2	2	
GPS	0	266 (86.4%)	165 (93.2%)	101 (77.1%)	< 0.001
	1–2	42 (13.6%)	12 (6.8%)	30 (22.9%)	
mGPS	0	278 (90.3%)	166 (93.8%)	112 (85.5%)	0.019
	1–2	30 (9.7%)	11 (6.2%)	19 (14.5%)	

After surgery					
Marker	Category	Total (*N* = 308)	AC group (*n* = 177)	Non‐AC group (*n* = 131)	*p*
NLR		1.90 (0.57–13.50)	1.92 (0.58–7.61)	1.86 (0.57–13.50)	0.215
LMR		5.31 (1.14–22.00)	5.50 (1.20–22.00)	5.07 (1.14–15.00)	0.079
PLR		134.3 (24.8–662.5)	133.1 (48.4–463.5)	137.0 (24.8–662.5)	0.337
CAR		0.025 (0.002–3.213)	0.024 (0.002–3.091)	0.025 (0.005–3.213)	0.012
PNI		48.2 (22.2–63.4)	48.7 (22.2–62.9)	47.4 (33.6–63.4)	0.003
GNRI		101.9 (59.9–126.6)	102.7 (59.9–126.6)	100.6 (77.8–121.1)	< 0.001
CONUT score	0–1	116 (59.2%)	70 (63.6%)	46 (53.5%)	0.311
	2–4	74 (37.8%)	37 (33.6%)	37 (43.0%)	
	≥ 5	6 (3.0%)	3 (2.8%)	3 (3.5%)	
	Unknown	112	67	45	
GPS	0	272 (88.3%)	163 (92.1%)	109 (83.2%)	0.020
	1–2	36 (11.7%)	14 (7.9%)	22 (16.8%)	
mGPS	0	287 (93.2%)	168 (94.9%)	119 (90.8%)	0.176
	1–2	21 (6.8%)	9 (5.1%)	12 (9.2%)	

*Note:* Data are presented as the number (percentage) or median (range) as appropriate.

Abbreviations: AC, adjuvant chemotherapy; CAR, C‐reactive protein‐to‐albumin ratio; CONUT, Controlling Nutritional Status; GNRI, Geriatric Nutritional Risk Index; GPS, Glasgow Prognostic Score; LMR, lymphocyte‐to‐monocyte ratio; mGPS, modified GPS; NLR, neutrophil‐to‐lymphocyte ratio; PLR, platelet‐to‐lymphocyte ratio; PNI Prognostic Nutritional Index.

For RFS, the preoperative cut‐off values and area under the curve for NLR, LMR, PLR, CAR, PNI, and GNRI were determined to be 2.63 (0.589), 5.25 (0.559), 156.2 (0.572), 0.027 (0.580), 49.0 (0.576), and 102.2 (0.527), respectively, with postoperative values of 1.98 (0.570), 5.27 (0.517), 135.6 (0.547), 0.026 (0.570), 47.9 (0.577), and 102.2 (0.527). Similarly, for OS, the preoperative cut‐off values and area under the curve for NLR, LMR, PLR, CAR, PNI, and GNRI were determined to be 2.61 (0.578), 5.33 (0.551), 156.2 (0.576), 0.027 (0.610), 49.0 (0.588), and 102.2 (0.553), respectively, with respective postoperative NLR, LMR, PLR, CAR, PNI, and GNRI values of 1.99 (0.557), 5.27 (0.521), 135.6 (0.548), 0.026 (0.568), 48.3 (0.582), and 102.2 (0.553). For the CONUT score, patients were compared in two ways by dividing them according to whether the score was ≤ 1 or ≥ 2. For GPS and modified GPS, patients were separated into those with a score of 0 and those with a score of 1 or 2.

### 3.5 Subgroup Analysis for Immuno‐Nutritional and Inflammatory Markers

**FIGURE 3:**
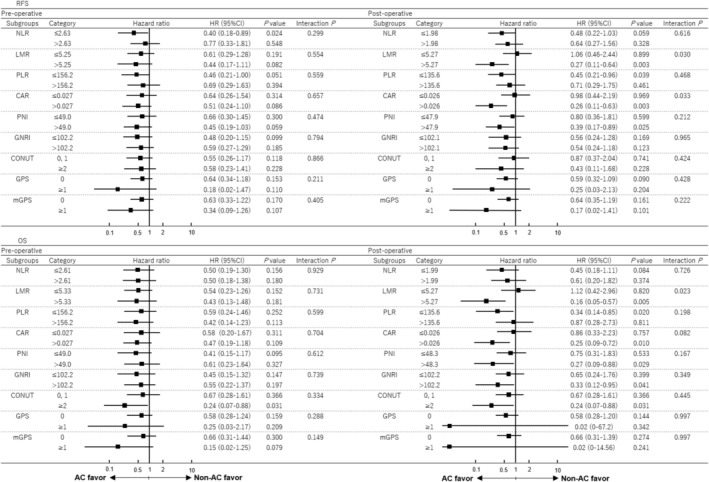
Hazard ratio for the effect of postoperative adjuvant chemotherapy on survival for each marker subgroup according to whether patients did or did not receive adjuvant chemotherapy. Each cut‐off value is listed. The *p*‐values were calculated by univariable Cox proportional hazard regression analysis. The interaction *p*‐value for each subgroup was calculated by multivariable Cox proportional hazard regression analysis. *AC* adjuvant chemotherapy, *CAR* C‐reactive protein‐to‐albumin ratio, CI confidence interval, *CONUT* Controlling Nutritional Status, *GNRI* Geriatric Nutritional Risk Index, *GPS* Glasgow Prognostic Score, HR hazard ratio, *LMR* lymphocyte‐to‐monocyte ratio, *mGPS* modified GPS, *NLR* neutrophil‐to‐lymphocyte ratio, *PLR* platelet‐to‐lymphocyte ratio, *PNI* Prognostic Nutritional Index.

After PSM, we performed a subgroup analysis to assess the interaction effects between immuno‐nutritional and inflammatory markers and adjuvant chemotherapy on survival outcomes. The patients were separated into subgroups according to whether the level of each marker was high or low. Pre‐ and postoperative HRs and *p*‐values for the effect of the markers on RFS and OS were calculated for each subgroup and summarized in the form of forest plots with interaction *p*‐values (Figure 3). The following factors were found to have an effect on the interaction of adjuvant chemotherapy with RFS and OS: postoperative LMR (interaction *p* = 0.030 and interaction *p* = 0.023, respectively) and postoperative CAR (interaction *p* = 0.033 and interaction *p* = 0.082).

**FIGURE 4:**
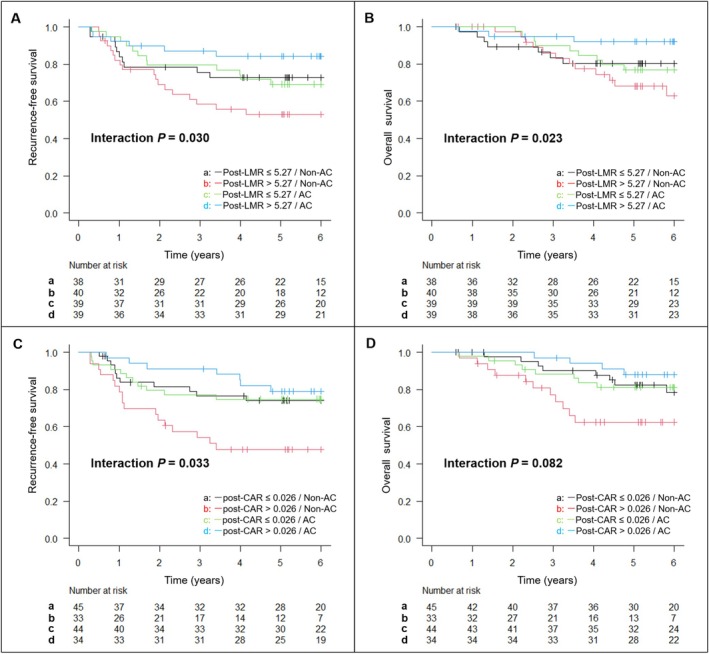
Kaplan–Meier survival curve stratified by each nutritional and inflammatory marker after propensity score matching according to whether postoperative adjuvant chemotherapy was administered. (A) RFS × postoperative LMR. (B) OS × postoperative LMR. (C) RFS × postoperative CAR. (D) OS × postoperative CAR. *AC* adjuvant chemotherapy, *CAR* C‐reactive protein‐to‐albumin ratio, *LMR* lymphocyte‐to‐monocyte ratio, *OS* overall survival, *Post*, postoperative, *RFS*, recurrence‐free survival.

In the subgroup with a high postoperative LMR, the RFS curve was significantly better for the AC group than for the non‐AC group (5‐year RFS rate: 84.4% vs. 53.0%; HR 0.27, 95% CI 0.11–0.64, *p* = 0.003). However, in the subgroup with a low postoperative LMR, the survival curve for the AC group was similar to that for the non‐AC group (5‐year RFS rate: 69.2% vs. 72.9%; HR 1.06, 95% CI 0.46–2.44, *p* = 0.899) (Figure 4A). A similar trend was also observed for OS. In the subgroup with a high postoperative LMR, the survival curve was better for the AC group than for the non‐AC group (5‐year OS rate: 92.1% vs. 68.0%; HR 0.16, 95% CI 0.05–0.57, *p* = 0.005). In the subgroup with a low postoperative LMR, the survival curves for the AC group and non‐AC group were similar (5‐year OS rate: 76.9% vs. 80.4%; HR 1.12, 95% CI 0.42–2.96, *p* = 0.820) (Figure 4B).

A similar trend was also observed for RFS and OS in relation to the postoperative CAR. In the subgroup with a high postoperative CAR, the survival curves for the AC group were better than those for the non‐AC group (5‐year RFS rate: 79.0% vs. 47.8%, HR 0.26, 95% CI 0.11–0.63, *p* = 0.003; 5‐year OS rate: 88.0% vs. 62.4%, HR 0.25, 95% CI 0.09–0.72, *p* = 0.010). In the subgroup with a low postoperative CAR, the survival curves for the AC group were similar to those for the non‐AC group (5‐year RFS rate, 74.8% vs. 74.2%, HR 0.98, 95% CI 0.44–2.19, *p* = 0.969; 5‐year OS rate, 81.2% vs. 82.4%, HR 0.86, 95% CI 0.33–2.23, *p* = 0.757) (Figure 4C,D).

## 4 Discussion

This study examined whether immuno‐nutritional and inflammatory markers can predict survival outcomes after postoperative adjuvant chemotherapy in patients aged ≥ 70 years after radical resection of stage III CRC when adjusted for patient background characteristics using PSM. Subgroup analysis after PSM identified the postoperative LMR to have a significant interaction with RFS and OS and the postoperative CAR to have a significant interaction with RFS. Postoperative LMR and CAR effectively stratified patients into high‐ and low‐value groups, with patients in the high‐value groups deriving significant survival benefits from AC, whereas the benefit was limited in those in the low‐value groups. To our knowledge, this is the first study to evaluate the prognostic impact of the interaction between immuno‐nutritional markers and AC specifically in elderly patients (≥ 70 years) with stage III CRC. The novelty of this work lies in its comprehensive analysis of multiple markers using both pre‐ and postoperative data within the same elderly cohort.

Before PSM, we found that adjuvant chemotherapy had a beneficial effect on both RFS (HR 0.495, *p* < 0.001) and OS (HR 0.397, *p* < 0.001). As recommended in various guidelines, adjuvant chemotherapy should be considered for elderly patients when appropriate. However, given the risk of complications, it is important to select those who are likely to derive a true benefit.

The markers identified as having an impact on survival in this study were the postoperative LMR (interaction *p* = 0.030 for RFS, interaction *p* = 0.023 for OS) and the postoperative CAR (interaction *p* = 0.033 for RFS, interaction *p* = 0.082 for OS), although the association between CAR and OS did not reach statistical significance (Figure 4). In patients with a low postoperative LMR, administration of adjuvant chemotherapy may not affect long‐term outcomes and could be omitted, but in those with a high postoperative LMR, adjuvant chemotherapy may significantly improve RFS and OS. The same applies to CAR. Our postoperative data identified several other potentially useful markers. Although these markers did not reach an interaction *p* < 0.05, survival benefits were identified when the patients were grouped according to adjuvant chemotherapy (Figure 3).

Cancer progression depends on the interplay between tumor characteristics and the host's immune‐inflammatory response. Local lymphocyte infiltration inhibits tumor progression, whereas systemic inflammation—characterized by increased neutrophils and exhausted T cells—correlates with poor outcomes [23, 24]. In this study, postoperative markers reflecting lymphocyte counts (NLR, PLR, PNI) showed similar trends to LMR, suggesting that patients who maintain postoperative immune competence derive greater benefit from adjuvant chemotherapy. This is consistent with findings in advanced CRC, suggesting that preservation of lymphocyte counts is associated with improved drug responsiveness [9].

CAR is the ratio of CRP to albumin, both of which are produced by hepatocytes and regulated by inflammatory cytokines such as interleukin‐1, interleukin‐6, and tumor necrosis factor‐*α*; it tends to increase under inflammatory conditions [25]. A previous study reported that a high postoperative CAR is an independent adverse prognostic factor for RFS and OS in stage III CRC, while also identifying a survival benefit from adjuvant chemotherapy [13]. Our results were consistent with these findings, despite restricting the analysis to elderly patients.

Regarding the timing of assessment, postoperative markers may be more informative than preoperative ones because adjuvant chemotherapy is administered after tumor removal. While preoperative values primarily reflect tumor‐driven chronic inflammation and can be confounded by factors like tumor obstruction, postoperative values reflect the host's intrinsic baseline immunity and the response to micrometastases [26, 27, 28]. Although surgery induces acute stress, inflammatory responses typically subside within 1 month [29]. In the present study, postoperative blood samples were collected uniformly 4–8 weeks after surgery, minimizing the impact of acute surgical inflammation. These markers were measured immediately before adjuvant chemotherapy, thereby reflecting the host's physiological and immunological status at treatment initiation.

The evidence for postoperative adjuvant chemotherapy in elderly patients with colorectal cancer remains limited, largely due to the exclusion of patients with comorbidities from clinical trials and small sample sizes in subgroup analyses, making extrapolation to real‐world clinical practice challenging [4, 30]. Although retrospective in nature, the present study included elderly patients with various common comorbidities and therefore reflects a population that is closer to real‐world clinical practice, which constitutes an important clinical strength. In recent years, circulating tumor DNA has been reported to be potentially useful for predicting the efficacy of adjuvant chemotherapy and for treatment monitoring; however, its high cost and issues related to generalizability continue to limit its widespread implementation in routine clinical practice [31]. In contrast, the postoperative immuno‐nutritional and inflammatory markers identified in this study can be calculated from routine blood tests at low cost and with minimal burden. At the time when postoperative pathological findings are finalized and the indication for adjuvant chemotherapy is considered, these markers may be used as supplementary reference information in addition to conventional clinical factors such as age, performance status, and comorbidities. Particularly in patients aged ≥ 70 years, in whom tolerability and survival benefit from adjuvant chemotherapy vary substantially among individuals, these markers may serve as adjunctive tools to help further individualize patient selection rather than to uniformly determine treatment indications.

This study has several limitations. First, because of the retrospective, single‐center nature of this study, selection bias and the influence of historical treatment practices on the choice of adjuvant chemotherapy could not be completely excluded, and the derived cut‐off values may reflect institution‐specific characteristics. Second, although propensity score matching was applied, complete balance across all covariates could not be achieved. Third, because this study was a retrospective analysis based on a clinical database, standardized assessment tools for comorbidities, such as the Charlson Comorbidity Index and Geriatric‐8, were not consistently available for all patients, particularly in older cases. Similarly, incomplete data limited the availability of detailed information on chemotherapy completion rates, dose reductions, and treatment‐related adverse events. Finally, information on RAS, BRAF, and MSI status is lacking because of the amount of older data included in the study.

## 5 Conclusion

Postoperative LMR and CAR values are useful predictors of the efficacy of postoperative adjuvant chemotherapy in elderly patients with stage III CRC and are expected to serve as potential markers to help inform decisions regarding adjuvant chemotherapy. Immuno‐nutritional and inflammatory markers have the potential to be utilized further in this field, and it is hoped that this research will lead to development of novel biomarkers for prediction of survival outcomes.

## Author Contributions

**Tomoya Tago:** conceptualization, data curation, methodology, formal analysis, investigation, writing – original draft, writing – review and editing. **Yasuyuki Takamizawa:** conceptualization, data curation, methodology, formal analysis, writing – original draft, writing – review and editing. **Takeharu Kato:** conceptualization, formal analysis, writing – review and editing. **Hiroshi Nagata:** conceptualization, methodology, formal analysis, writing – review and editing. **Konosuke Moritani:** conceptualization, formal analysis, writing – review and editing. **Shunsuke Tsukamoto:** conceptualization, formal analysis, writing – review and editing, supervision. **Yukihide Kanemitsu:** conceptualization, formal analysis, writing – review and editing, supervision.

## Funding

The authors have nothing to report.

## Ethics Statement

This study was approved by the Institutional Review Board of the National Cancer Center Hospital (approval no. 2017–437). The requirement for written informed consent was waived because this study involved only retrospective analysis of de‐identified data. This study was conducted in accordance with the Declaration of Helsinki.

## Consent

The requirement for written informed consent was waived because this study involved only retrospective analysis of de‐identified data.

## Conflicts of Interest

The authors declare no conflicts of interest.

## Data Availability

The data that support the findings of this study are available from the corresponding author upon reasonable request.
